# Work Performance Challenges and Needs of Adults with ADHD: Exploring Lived Experiences

**DOI:** 10.1192/j.eurpsy.2025.2169

**Published:** 2025-08-26

**Authors:** N. Grinblat, S. Rosenblum

**Affiliations:** 1 Department of Occupational Therapy, University of Haifa, Haifa, Israel

## Abstract

**Introduction:**

Attention-deficit hyperactivity disorder (ADHD) significantly impacts adults’ work performance, yet their lived experiences and perceptions of their work challenges remain underexplored. Understanding these subjective viewpoints is crucial for capturing the complexities of this population’s daily work challenges and needs, serving as a foundational step in developing targeted intervention to enhance work performance and participation.

**Objectives:**

This study aimed to explore the work performance experiences, challenges, and needs of adults with ADHD.

**Methods:**

Twelve adults (ages 20-46) diagnosed with ADHD participated in three separate online focus groups, each comprising four participants.

**Results:**

Participants reported key challenges in executive functions, including difficulties with time management (lateness, missing deadlines), planning (prioritizing tasks, multitasking), working memory (forgetting instructions and names), maintaining focus, managing distractions, and emotional regulation (struggles in relationships with colleagues and employers). These challenges often led to frustration, stress, and low occupational self-efficacy. Identified needs included psychoeducation, self-regulation strategies, work and environment accommodations, and personalized interventions.

**Conclusions:**

The study highlights the unique executive challenges and emotional consequences faced by working adults with ADHD. Hence, it emphasizes the need for personalized interventions to enhance work performance, participation, and overall well-being in this population.

**Disclosure of Interest:**

None Declared

